# Racial Disparities in Patient-Provider Communication During Telehealth Visits Versus Face-to-face Visits Among Asian and Native Hawaiian and Other Pacific Islander Patients With Cancer: Cross-sectional Analysis

**DOI:** 10.2196/37272

**Published:** 2022-12-09

**Authors:** Jared D Acoba, Chelsea Yin, Michael Meno, Justin Abe, Ian Pagano, Sharon Tamashiro, Kristy Fujinaga, Christa Braun-Inglis, Jami Fukui

**Affiliations:** 1 University of Hawaii Cancer Center Honolulu, HI United States; 2 Kaiser Permanente Oakland, CA United States; 3 University of Washington Seattle, WA United States; 4 University of Southern California Los Angeles, CA United States; 5 Queen's Medical Center Honolulu, HI United States

**Keywords:** cancer, telemedicine, telehealth, eHealth, racial disparities, race, racial, Asia, Asian, Hawaii, Hawaiian, Native Hawaiian, Pacific Islander, cross-sectional, satisfaction, cancer, oncology, racially diverse, patient-physician communication

## Abstract

**Background:**

Telehealth visits increase patients’ access to care and are often rated as “just as good” as face-to-face visits by oncology patients. Telehealth visits have become increasingly more common in the care of patients with cancer since the advent of the COVID-19 pandemic. Asians and Pacific Islanders are two of the fastest growing racial groups in the United States, but there are few studies assessing patient satisfaction with telemedicine among these two racial groups.

**Objective:**

Our objective was to compare satisfaction with communication during telehealth visits versus face-to-face visits among oncology patients, with a specific focus on Asian patients and Native Hawaiian and other Pacific Islander (NHOPI) patients.

**Methods:**

We surveyed a racially diverse group of patients who were treated at community cancer centers in Hawaii and had recently experienced a face-to-face visit or telehealth visit. Questions for assessing satisfaction with patient-physician communication were adapted from a previously published study of cancer survivors. Variables that impact communication, including age, sex, household income, education level, and cancer type and stage, were captured. Multivariable logistic models for patient satisfaction were created, with adjustments for sociodemographic factors.

**Results:**

Participants who attended a face-to-face visit reported higher levels of satisfaction in all communication measures than those reported by participants who underwent a telehealth encounter. The univariate analysis revealed lower levels of satisfaction during telehealth visits among Asian participants and NHOPI participants compared to those among White participants for all measures of communication (eg, when asked to what degree “[y]our physician listened carefully to you”). Asian patients and NHOPI patients were significantly less likely than White patients to strongly agree with the statement (*P*<.004 and *P*<.007, respectively). Racial differences in satisfaction with communication persisted in the multivariate analysis even after adjusting for sociodemographic factors. There were no significant racial differences in communication during face-to-face visits.

**Conclusions:**

Asian patients and NHOPI patients were significantly less content with patient-physician communication during telehealth visits when compared to White patients. This difference among racial groups was not seen in face-to-face visits. The observation that telehealth increases racial disparities in health care satisfaction should prompt further exploration.

## Introduction

Telehealth is the use of real-time audio and video technologies for telecommunication between patients and health care providers. Telehealth visits increase patients’ access to care by reducing travel time and expenses and by providing increased schedule flexibility. Telehealth also allows health care providers to reach patients and other specialists remotely, allows them to reach larger segments of the population, alleviates workforce shortages in remote areas, and improves care coordination [1-6]. Patient satisfaction with telehealth has been well documented, particularly among residents from rural communities [2,4,6-8], with as many as 95% of patients rating telehealth visits as “better than” or “just as good” as face-to-face visits [6,9,10]. Specifically, studies of oncology patients have reported high levels of satisfaction with telehealth [3-5,8,11-14].

With the advent of the COVID-19 pandemic, the American Society of Clinical Oncology released guidelines that advocate for the use of telemedicine for patients not requiring face-to-face services, such as physical examinations, treatments, and in-office diagnostics [15]. In response, oncology practices increased the number of telehealth visits to reduce the risk of SARS-CoV-2 transmission [3,13,14,16]. Teleoncology studies that were conducted during the COVID-19 pandemic demonstrated that telehealth visits met the needs of oncology patients, without a reduction in services [14,17].

As the use of telehealth increases, it is important to ensure that this care modality is beneficial to all patients with cancer. Numerous studies have shown the lower use of telehealth among racial minority patients [18-21]. Chunara et al [20] demonstrated that while Black individuals increased their use of telehealth during the early part of the COVID-19 pandemic, their use remained lower than that of their White counterparts. Hiratsuka et al [21] noted that Native Hawaiian and Alaska Native patients see the “lack of physical contact and hands-on interaction” as a disadvantage of telehealth visits.

There is a paucity of literature evaluating patient satisfaction and the quality of communication during telehealth encounters among Asian patients and Native Hawaiian and other Pacific Islander (NHOPI) patients. Asians and Pacific Islanders are two of the fastest growing racial groups in the United States [22,23], and cancer incidence and mortality rates are higher among patients belonging to these groups than those among White patients [24]. Assessing Asian patients’ and NHOPI patients’ interactions with health care providers in telemedicine encounters could prove valuable. Our objective was to compare satisfaction with communication during telehealth visits versus face-to-face visits among oncology patients, with a specific focus on Asian patients and NHOPI patients.

## Methods

### Participants and Eligibility Criteria

This study compared survey responses from a racially diverse group of patients with cancer who were treated at community cancer centers in Hawaii. Patients with cancer aged ≥18 years were eligible, and participants needed to be able to communicate in English without the assistance of a translator.

#### Face-to-face Survey

We assessed patient satisfaction with communication during face-to-face visits by surveying patients who underwent survivorship care visits from January 2014 through June 2018 at the Queen’s Cancer Center (Honolulu, Hawaii). These cancer survivors had received definitive cancer therapy with curative intent and were invited to complete the survey during a period of follow-up care. We mailed eligible participants invitations to the survey and collected survey responses via the internet or over the phone from September 2018 through December 2018.

#### Telehealth Survey

To gauge satisfaction during telehealth visits, we surveyed patients who experienced a telehealth visit between March 2020 and August 2020 at outpatient cancer centers in Hawaii that were affiliated with the Queen’s Cancer Center and Hawaii Pacific Health (Honolulu). Eligible participants included patients who were actively receiving treatment with either curative or palliative intent and patients in follow-up care. We approached participants of the telehealth survey sequentially within the survey time frame and invited them to participate in the survey either by phone or via the internet.

### Data Collection and Measurement

All face-to-face and telehealth surveys were completed anonymously, and no personal health information or personally identifiable information was collected. The demographic data collected included sex, age, education level, household income, insurance type, race, the type of cancer, and the stage of cancer. Age was categorized as <50 years, 50 to 59 years, 60 to 79 years, and ≥80 years. Education levels were grouped into the following five categories: high school degree or less, some college but no formal degree, associate’s or bachelor’s degree, master’s or doctorate degree, and other. Classifications for household income included “prefer not to say,” <US $30,000 per year, US $30,000 to US $59,999 per year, US $60,000 to US $89,999 per year, and ≥US $90,000 per year. Patients self-identified a single race that best described them and were grouped as White, NHOPI, Asian, or other race patients. Cancers were clustered as gastrointestinal cancer (colon cancer, cholangiocarcinoma, hepatoma, gastric cancer, or esophageal cancer); hematopoietic cancer (acute myeloid leukemia, myelodysplastic syndrome, lymphoma, or myeloma); genitourinary cancer (prostate, bladder, or kidney cancer); gynecologic cancer (ovarian or uterine cancer); breast cancer; lung, head, and neck cancer; or other. Cancer stages were grouped as “I do not remember,” stage 0 to 2, and stage 3 to 4.

Questions for assessing communication were adapted from a previous study of cancer survivors by Palmer et al [25]. These questions were part of the Assessment of Patient Experiences of Cancer Care (APECC) study [26], which included questions from existing surveys and items developed by the APECC investigators. Patients were asked to rate their degree of agreement with the following eight statements regarding communication with their physician: (1) “Your physician listened carefully to you,” (2) “Your physician explained things in a way you could understand,” (3) “Your physician showed respect for what you had to say,” (4) “Your physician encouraged you to ask all of the cancer-related questions you had,” (5) “Your physician made sure that you understood all of the information he or she gave you,” (6) “Your physician spent enough time with you,” (7) “Your physician gave you as much cancer-related information as you wanted,” and (8) “Your physician involved you in decisions about your medical care as much as you wanted.” Responses were assessed on a 5-point response scale ranging from “strongly disagree” to “strongly agree.”

### Outcomes

The main outcomes of interest were (1) the degree to which patients agreed that their health care provider met the measures of communication described in the *Data Collection and Measurement* section and (2) whether the ratings for communication varied significantly by race.

### Statistical Methods

To avoid issues of nonnormality and to ensure that the methods used to analyze all variables were consistent, continuous demographic variables were grouped into categories, and chi-square tests were used to assess differences across groups. A *P* value of <.05 was considered statistically significant. The degree of patient satisfaction was analyzed by comparing patients who strongly agreed with statements to those who submitted other answers. Multivariable logistic models for patient satisfaction were built to obtain odds ratios (ORs) and 95% CIs, adjusting for sociodemographic factors. SPSS version 27.0 (IBM Corporation) was used for all analyses.

### Ethics Approval

This study was approved by the Queen’s Medical Center and Hawaii Pacific Health research and institutional review committees (approval numbers: RA-2020-20 and RA-2018-038).

## Results

### Patient Population

A total of 593 surveys were collected, with 362 participants in the face-to-face group (response rate: 362/1419, 25.5%) and 231 in the telehealth group (response rate: 231/464, 49.8%). Baseline demographics, including sex (*P*=.79), age (*P*=.10), education level (*P*=.15), household income (*P*=.82), and race (*P*=.41), did not differ significantly between the two groups (Table 1). Participants were highly educated, with the majority (479/587, 81.6%) having some college or more education. There were more cases of gynecologic cancers and head, neck, and lung cancer among the face-to-face group respondents and more cases of gastrointestinal and hematologic cancers among the telehealth group participants (*P*<.001). The majority (240/362, 66.3%) of the face-to-face group reported earlier cancer stages than those reported by the telehealth group.

**Table 1:** Baseline characteristics (N=593).

Characteristic			Face-to-face group (n=362), n (%)		Telehealth group (n=231), n (%)		*P* value	
**Sex**								.79
	Female	240 (66.3)		150 (64.9)				
	Male	122 (33.7)		81 (35.1)				
**Age (years)**								.10
	<50	23 (6.4)		27 (11.7)				
	50-59	58 (16.1)		42 (18.3)				
	60-69	137 (38.1)		77 (33.5)				
	≥70	142 (39.4)		84 (36.5)				
**Education**								.15
	High school degree or less	52 (14.6)		37 (16.1)				
	Some college	82 (23)		40 (17.4)				
	Associate’s or bachelor’s degree	139 (38.9)		105 (45.7)				
	Master’s or doctorate degree	75 (21)		38 (16.5)				
	Other	9 (2.5)		10 (4.3)				
**Household income per year (US $)**								.82
	<30,000	48 (13.3)		37 (16)				
	30,000-59,999	64 (17.7)		35 (15.2)				
	60,000-89,999	69 (19.1)		44 (19)				
	≥90,000	117 (32.3)		71 (30.7)				
	Prefer not to say	64 (17.7)		44 (19)				
**Race**								.41
	White	87 (24.4)		51 (22.6)				
	Asian	213 (59.8)		127 (56.2)				
	Native Hawaiian and other Pacific Islander	41 (11.5)		36 (15.9)				
	Other	15 (4.2)		12 (5.3)				
**Cancer type**								<.001
	Breast	132 (36.5)		101 (43.7)				
	Lung, head, and neck	64 (17.7)		21 (9.1)				
	Genitourinary	54 (14.9)		22 (9.5)				
	Gastrointestinal	32 (8.8)		53 (22.9)				
	Gynecologic	46 (12.7)		0 (0)				
	Hematologic	11 (3)		25 (10.8)				
	Other^a^	23 (6.4)		9 (3.9)				
**Cancer stage**								<.001
	0-1	151 (41.7)		69 (29.9)				
	2	89 (24.6)		37 (16)				
	3-4	70 (19.3)		74 (32)				
	Unsure	52 (14.4)		51 (22.1)				

^a^Includes melanoma, Merkel cell carcinoma, sarcoma, thyroid cancer, and unknown primary cancer.

### Face-to-face Visits Versus Telehealth Visits

**Figure 1:**
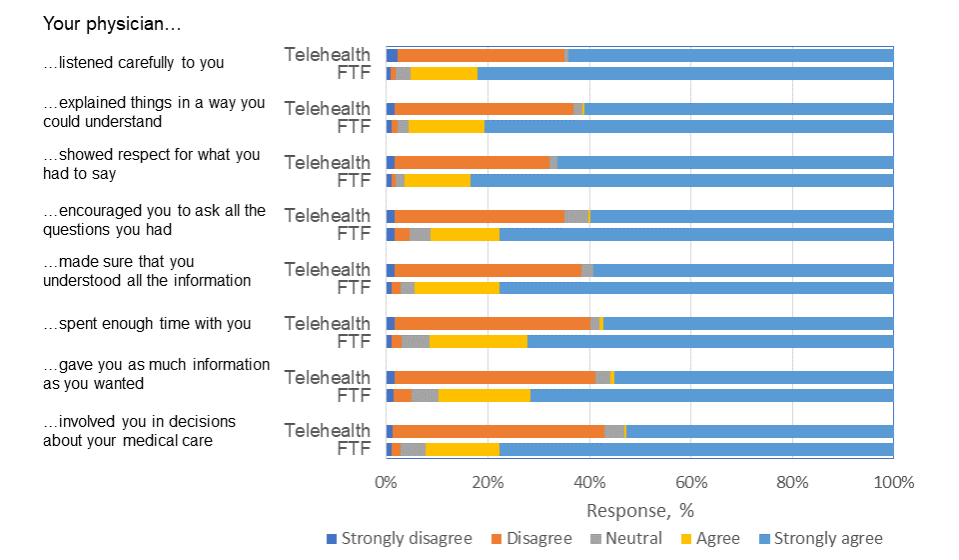
Satisfaction among the telehealth and FTF groups. FTF: face-to-face.

Participants who attended a face-to-face visit reported higher levels of satisfaction in all communication measures (all *P* values were <.05) than those reported by participants who experienced a telehealth encounter (Figure 1).

Logistic regression models were created to measure the association between patient demographics and satisfaction with patient-physician communication. The univariate analysis revealed significant racial differences in the telehealth group but not in the face-to-face group. For example, White patients were more likely to strongly agree with the statement “Your physician listened carefully to you” (Table 2) than Asian patients (OR 0.26, 95% CI 0.10-0.6) and NHOPI patients (OR 0.20, 95% CI 0.06-0.64).

Table 2 illustrates the degree to which patients agreed that their physicians listened carefully to them by characteristic. Degrees of satisfaction were divided into 2 groups—the strongly agree and other answers groups—to calculate an OR.

Similar racial disparities were detected for each of the eight communication statements (Table 3). Asian patients and NHOPI patients were significantly less likely to be satisfied with patient-physician communication during telehealth visits when compared to White patients. This difference was not seen in face-to-face visits.

Table 3 illustrates the results of a univariate analysis of the degree to which patients agreed that their health care provider met measures of satisfaction by race. Degrees of satisfaction were divided into 2 groups—the strongly agree and other answers groups—to calculate an OR. Participants who selected “White” as their primary ethnicity were used as the reference group.

Differences in racial perceptions of communication during telehealth visits persisted in a multivariate analysis even after adjusting for age, sex, household income, education level, and cancer type and stage (Table 4). In contrast, there were no significant racial differences in communication during face-to-face visits.

Table 4 illustrates the results of a multivariate analysis of the degree to which patients agreed that their health care provider met measures of satisfaction by race. Degrees of satisfaction were divided into 2 groups—the strongly agree and other answers groups—to calculate an OR. Sex, age, education, and household income were factored into the regression model. Participants who selected “White” as their primary ethnicity were used as the reference group.

**Table 2:** Univariate analysis of results for the statement “Your physician listened carefully to you.”

Characteristic			Face-to-face group					Telehealth group		
			Odds ratio (95% CI)		*P* value		Odds ratio (95% CI)			*P* value
**Sex**										
	Male (reference)	N/A^a^		N/A		N/A			N/A	
	Female	0.80 (0.27-2.34)		.68		2.58 (1.02-6.51)			.045^b^	
**Age (years)**										
	<50 (reference)	N/A		N/A		N/A			N/A	
	50-59	0.46 (0.10-2.05)		.31		0.40 (0.12-1.34)			.14	
	60-69	0.68 (0.16-2.91)		.60		0.46 (0.16-1.31)			.15	
	≥70	1.43 (0.32-6.36)		.64		0.72 (0.23-2.31)			.58	
**Education**										
	High school degree or less (reference)	N/A		N/A		N/A			N/A	
	Some college	1.83 (0.69-4.88)		.23		3.04 (1.06-8.78)			.04^b^	
	Associate’s or bachelor’s degree	1.41 (0.55-3.58)		.47		1.60 (0.62-4.10)			.33	
	Master’s or doctorate degree	1.64 (0.55-4.83)		.37		3.14 (0.92-10.79)			.07	
**Household income per year (US $)**										
	<30,000 (reference)	N/A		N/A		N/A			N/A	
	30,000-59,999	4.50 (1.30-15.65)		.02^b^		0.33 (0.11-1.02)			.053	
	60,000-89,999	0.95 (0.33-2.73)		.92		1.04 (0.35-3.12)			.95	
	≥90,000	1.79 (0.62-5.21)		.29		0.82 (0.30-2.25)			.70	
**Race**										
	White (reference)	N/A		N/A		N/A			N/A	
	Asian	1.19 (0.54-2.64)		.66		0.26 (0.10-0.64)			.004^b^	
	Native Hawaiian and other Pacific Islander	1.33 (0.43-4.16)		.63		0.20 (0.06-0.64)			.007^b^	
**Cancer type**										
	Breast (reference)	N/A		N/A		N/A			N/A	
	Lung, head and neck	1.92 (0.55-6.68)		.31		2.85 (0.73-11.20)			.13	
	Genitourinary and prostate	4.05 (0.88-18.61)		.07		2.27 (0.47-10.97)			.31	
	Gastrointestinal	1.77 (0.46-6.81)		.41		1.61 (0.57-4.56)			.37	
	Endometrial and ovarian	0.41 (0.17-0.97)		.04^b^		N/A			N/A	
	Blood­	0.54 (0.12-2.39)		.42		1.52 (0.42-5.51)			.53	
**Cancer stage**										
	0-1 (reference)	N/A		N/A		N/A			N/A	
	2	0.89 (0.37-2.03)		.74		0.73 (0.28-1.86)			.51	
	3-4	0.37 (0.15-0.90)		.03^b^		0.75 (0.31-1.81)			.52	
	Unsure	0.39 (0.13-1.16)		.44		0.86 (0.30-2.44)			.78	

^a^N/A: not applicable.

^b^Significant at the *P*<.05 level.

**Table 3:** Univariate analysis.

Statement and race		Face-to-face group			Telehealth group	
		Odds ratio (95% CI)	*P* value	Odds ratio (95% CI)		*P* value
“**Your physician listened carefully to you”**						
	Asian	1.19 (0.54-2.64)	.66	0.26 (0.10-0.64)		.004^a^
	NHOPI^b^	1.33 (0.43-4.16)	.63	0.20 (0.06-0.64)		.007^a^
“**Your physician explained things in a way you could understand”**						
	Asian	0.87 (0.46-1.64)	.66	0.27 (0.12-0.61)		.003^a^
	NHOPI	1.10 (0.41-2.91)	.86	0.21 (0.08-0.57)		.008^a^
“**Your physician showed respect for what you had to say”**						
	Asian	1.02 (0.53-1.99)	.94	0.26 (0.11-0.63)		.003^a^
	NHOPI	1.01 (0.38-2.71)	.98	0.18 (0.06-0.50)		.005^a^
“**Your physician encouraged you to ask all of the cancer-related questions you had”**						
	Asian	0.97 (0.53-1.75)	.91	0.48 (0.23-0.98)		.04^a^
	NHOPI	1.27 (0.51-3.19)	.61	0.24 (0.10-0.61)		.004^a^
“**Your physician made sure that you understood all of the information he or she gave you”**						
	Asian	0.81 (0.44-1.49)	.49	0.46 (0.23-0.95)		.04^a^
	NHOPI	1.09 (0.43-2.77)	.85	0.31 (0.12-0.76)		.02^a^
“**Your physician spent enough time with you”**						
	Asian	0.76 (0.43-1.36)	.36	0.48 (0.24-0.97)		.04^a^
	NHOPI	0.88 (0.38-2.06)	.77	0.27 (0.11-0.67)		.01^a^
“**Your physician gave you as much cancer-related information as you wanted”**						
	Asian	0.64 (0.36-1.14)	.13	0.44 (0.22-0.88)		.03^a^
	NHOPI	0.73 (0.32-1.69)	.47	0.30 (0.12-0.73)		.02^a^
“**Your physician involved you in decisions about your medical care as much as you wanted”**						
	Asian	0.80 (0.43-1.51)	.50	0.45 (0.23-0.89)		.03^a^
	NHOPI	0.63 (0.18-2.23)	.10	0.33 (0.13-0.79)		.02^a^

^a^Significant at the *P*<.05 level.

^b^NHOPI: Native Hawaiian and other Pacific Islander.

**Table 4:** Multivariate analysis.

Statement and race			Face-to-face group				Telehealth group		
			Odds ratio (95% CI)		*P* value		Odds ratio (95% CI)		*P* value
“**Your physician listened carefully to you”**									
	Asian	1.25 (0.60-2.62)		.34		0.27 (0.11-0.65)		.004^a^	
	NHOPI^b^	1.39 (0.47-4.15)		.54		0.20 (0.06-0.64)		.007^a^	
“**Your physician explained things in a way you could understand”**									
	Asian	1.26 (0.60-2.62)		.44		0.26 (0.11-0.63)		.002^a^	
	NHOPI	1.62 (0.54-4.87)		.36		0.24 (0.08-0.76)		.01^a^	
“**Your physician showed respect for what you had to say”**									
	Asian	1.32 (0.60-2.88)		.36		0.25 (0.10-0.63)		.004^a^	
	NHOPI	1.28 (0.41-3.99)		.63		0.19 (0.06-0.64)		.005^a^	
“**Your physician encouraged you to ask all of the cancer-related questions you had”**									
	Asian	1.10 (0.56-2.19)		.69		0.46 (0.21-1.11)		.07	
	NHOPI	1.40 (0.50-3.93)		.49		0.23 (0.08-0.68)		.006^a^	
“**Your physician made sure that you understood all of the information he or she gave you”**									
	Asian	1.04 (0.52-2.07)		.43		0.49 (0.23-1.06)		.08	
	NHOPI	1.51 (0.53-4.33)		.88		0.35 (0.12-1.00)		.049^a^	
“**Your physician spent enough time with you”**									
	Asian	0.86 (0.45-1.63)		.75		0.47 (0.22-1.01)		.07	
	NHOPI	1.12 (0.43-2.89)		.83		0.29 (0.10-0.83)		.02^a^	
“**Your physician gave you as much cancer-related information as you wanted”**									
	Asian	0.71 (0.37-1.38)		.13		0.47 (0.22-0.99)		.049^a^	
	NHOPI	0.90 (0.35-2.35)		.47		0.38 (0.14-1.10)		.06	
“**Your physician involved you in decisions about your medical care as much as you wanted”**									
	Asian	0.96 (0.47-1.95)		.98		0.43 (0.20-0.92)		.04^a^	
	NHOPI	0.67 (0.25-1.78)		.43		0.40 (0.14-1.14)		.06	

^a^Significant at the *P*<.05 level.

^b^NHOPI: Native Hawaiian and other Pacific Islander.

## Discussion

### Principal Findings

Overall, patients with cancer in our racially diverse cohort were content with patient-physician communication. However, the patients who experienced telehealth visits were less satisfied than their counterparts who underwent face-to-face visits. Importantly, Asian patients and NHOPI patients were significantly less content with patient-physician communication during telehealth visits when compared to White patients—a disparity that was not evident in face-to-face visits.

The difference in satisfaction demonstrated between the two types of patient visits differs from the results of prior studies that demonstrated equivalent satisfaction with communication between face-to-face encounters and telehealth encounters [27,28]. In these prior studies, telehealth was an accepted alternative and was pursued due to the long distances between the patients’ homes and the clinics. In our telehealth group, lower ratings may have occurred because these patients viewed face-to-face visits as the standard of care and only converted to telehealth due to the COVID-19 pandemic. In addition, our face-to-face group consisted only of cancer survivors who had received definitive cancer therapy with curative intent, whereas our telehealth group included patients in follow-up care and those who were being actively treated with both curative intent and palliative intent. These differences may have adversely impacted perceptions of communication among the telehealth patients, as patients with a poor health status tend to report worse experiences [29].

Our study showed that Asian and Pacific Islander patients were significantly less satisfied with communication with their physicians during telehealth visits when compared to White patients. This racial disparity was not present in face-to-face visits and persisted even after adjusting for age, education level, and household income. Racial differences in perceptions of communication among patients with cancer have been previously reported. For instance, Asian cancer survivors have reported poorer follow-up communication and care quality [25] compared to those reported by White cancer survivors. Our study however is the first to demonstrate a racial disparity in communication exclusively for those who experienced telehealth visits. A study assessing telemedicine perspectives in Native Hawaiian and Alaska Native communities highlighted the need for a culturally appropriate telehealth approach. The focus groups stressed that a successful visit hinged on understanding the importance of the communication practices of racial minority patients, such as processing before speaking [21]. Methods of practicing culturally sensitive care during telehealth visits should be explored, given the increasing efforts to reduce barriers to telehealth for racial minority patients [30].

In contrast to other studies demonstrating racial disparities in communication [25], our study found no significant racial differences in the face-to-face setting. The higher level of satisfaction that we observed among racial minority patients may have been due to the difference in racial distribution between Hawaii and the continental United States. Hawaii is a majority-minority state, and racial minority patients and White patients with cancer receive care at the same clinical centers. The majority of cancer health care providers in Hawaii are also racial minority individuals, and racial concordance between patients and health care providers [31-33] has been shown to improve communication. It is conceivable that the oncology providers at Hawaii’s community cancer centers may display greater cultural competence when compared to the average oncology provider [34].

To our knowledge, this is the first study to differentiate Asian perceptions and NHOPI perceptions of communication in telehealth encounters. When asked about the time and encouragement given by their health care providers to ask questions during telehealth visits, NHOPI patients gave lower scores than those given by White patients. NHOPI patients have stressed that taking time to talk and verifying their understanding were ways to show genuine concern and care [21]. These steps may not have been taken, as telehealth was abruptly introduced not only to the patients but also to the health care providers, who may not have been aware of these particular NHOPI perceptions. Further, when asked about the information that they were given in telehealth visits and their involvement during these visits, Asian patients gave lower scores than those given by White patients, which is consistent with studies showing lower perceived self-efficacy and control over care among Asian patients [25]. Health care providers caring for Asian individuals and NHOPI individuals should be attentive to these communication disparities in telehealth visits.

### Limitations

This study has several strengths. The participants were treated at community cancer centers, which makes our findings generalizable to the majority of patients with cancer in the United States [35]. The majority of patients (455/593, 76.7%) comprising the study population were from racial minority groups who are typically underrepresented in cancer studies. Specifically, we incorporated a large number of NHOPI patients with cancer, for whom there are limited data on perceptions of communication and telemedicine. There are also limitations to our study. First, as stated above, the face-to-face group patients were all cancer survivors, per the definition provided by the Commission on Cancer [36], as they received definitive cancer therapy with a curative intent, while the telehealth patients included both cancer survivors and patients with cancer on active treatment. Second, the patients and health care providers viewed face-to-face visits as the norm and only converted to telehealth visits due to the COVID-19 pandemic. Although these differences may have affected the overall satisfaction levels of the two groups, they were not expected to account for the racial disparity seen exclusively in the telehealth group. Third, although we adapted our communication assessment from a previously published study [25], we did not use a validated communication assessment tool. However, we showed significant racial differences across a number of communication questions, and it is likely that a disparity would have been similarly demonstrated by a validated tool. Fourth, we did not capture information on English language proficiency. Although all eligible patients were able to communicate in English, it is conceivable that English being a second language was more prevalent among Asian patients and NHOPI patients than among White patients, and this could have impacted satisfaction with communication more greatly in telemedicine visits than in face-to-face visits [30].

### Conclusion

We present a study of patient-provider communication among a racially diverse population of patients with cancer that provides insight into racial disparities in telehealth visits that are not seen in face-to-face encounters. With the increasing popularity of telehealth, it is likely that telehealth visits will continue beyond the COVID-19 pandemic. Further investigation is needed to understand the strengths and limitations of telehealth and provide optimal care. The observation that telehealth increases racial disparities in health care satisfaction should prompt further exploration. An improved understanding of this issue will aid health care providers in making decisions about the delivery of care for their patients.

## Abbreviations

APECC: Assessment of Patient Experiences of Cancer Care
NHOPI: Native Hawaiian and other Pacific Islander
OR: odds ratio
